# Linking Functional Limitations and Relationship Satisfaction: The Role of Helping and Internet Use

**DOI:** 10.1093/geroni/igaf122.3620

**Published:** 2025-12-31

**Authors:** Debarati Kole, Heather Fuller

**Affiliations:** North Dakota State University, Fargo, North Dakota, United States; North Dakota State University, Fargo, North Dakota, United States

## Abstract

As people age, maintaining satisfying relationships is important for overall well-being; however, functional limitations can challenge relationship quality in part due to increased need for caregiving-related help and decreased ability to reciprocate that help. In today’s digital age, internet use may help older adults to stay engaged in giving and receiving help despite any functional limitations. The present study sought to understand: 1) Does the frequency of receiving and giving help to friends, family, and neighbors mediate the relationship between functional limitation and relationship satisfaction?, and 2) Does internet use play a moderating role? The data is drawn from the Social Integration and Aging Study (2013), a community-based survey of older adults (60+) in the Upper Midwest (N = 416). Bootstrap analyses were conducted with PROCESS macro models 4 and 58. Frequency of receiving help did not mediate the association between functional limitations and relationship satisfaction. However, frequency of giving help meditated the association between functional limitations and relationship satisfaction (a= -0.05***, b = 0.36***, c=-0.05***, c’=0.03**). Furthermore, moderated mediation was evident as frequency of internet use moderated the effect of helping others, indicating that those with greater functional limitations provide more help if they are internet users. Findings highlight the role of giving help to others for older adults to maintain satisfying relationships. The study also indicates that internet use can create opportunities for older adults to offer support. Future research should investigate these effects longitudinally and explore interventions to enhance giving and receiving support among older adults by improving their digital skills.

